# Mitochondrial DNA Mutations in Grade II and III Glioma Cell Lines Are Associated with Significant Mitochondrial Dysfunction and Higher Oxidative Stress

**DOI:** 10.3389/fphys.2017.00231

**Published:** 2017-04-21

**Authors:** Bee Hong Soon, Nor Azian Abdul Murad, Sue-Mian Then, Azizi Abu Bakar, Farizal Fadzil, Jegan Thanabalan, Mohd S. Mohd Haspani, Charng Jeng Toh, Azmi Mohd Tamil, Roslan Harun, Wan Z. Wan Ngah, Rahman Jamal

**Affiliations:** ^1^UKM Medical Molecular Biology Institute, Universiti Kebangsaan MalaysiaKuala Lumpur, Malaysia; ^2^Division of Neurosurgery, Department of Surgery, Faculty of Medicine, Universiti Kebangsaan MalaysiaKuala Lumpur, Malaysia; ^3^The University of Nottingham Malaysia CampusSemenyih, Malaysia; ^4^Neurosurgery Department, Hospital Kuala LumpurKuala Lumpur, Malaysia; ^5^Department of Community Health, Faculty of Medicine, Universiti Kebangsaan MalaysiaKuala Lumpur, Malaysia

**Keywords:** gliomas, mitochondrial DNA mutation, mitochondrial dysfunction, oxidative stress

## Abstract

The role of mitochondria in tumorigenesis has regained much attention as it could dysregulate cellular energetics, oxidative stress and apoptosis. However, the role of mitochondria in different grade gliomasis still unknown. This study aimed to identify mitochondrial DNA (mtDNA) sequence variations that could possibly affect the mitochondrial functions and also the oxidative stress status. Three different grades of human glioma cell lines and a normal human astrocyte cell line were cultured *in-vitro* and tested for oxidative stress biomarkers. Relative oxidative stress level, mitochondria activity, and mitochondrial mass were determined by live cell imaging with confocal laser scanning microscope using CM-H_2_DCFDA, MitoTracker Green, and MitoTracker Orange stains. The entire mitochondrial genome was sequenced using the AffymetrixGeneChip Human Mitochondrial Resequencing Array 2.0. The mitochondrial sequence variations were subjected to phylogenetic haplogroup assessment and pathogenicity of the mutations were predicted using pMUT and PolyPhen2. The Grade II astrocytoma cells showed increased oxidative stress wherea high level of 8-OHdG and oxidative stress indicator were observed. Simultaneously, Grade II and III glioma cells showed relatively poor mitochondria functions and increased number of mutations in the coding region of the mtDNA which could be due to high levels of oxidative stress in these cells. These non-synonymous mtDNA sequence variations were predicted to be pathogenic and could possibly lead to protein dysfunction, leading to oxidative phosphorylation (OXPHOS) impairment, mitochondria dysfunction and could create a vicious cycle of oxidative stress. The Grade IV cells had no missense mutation but preserved intact mitochondria and excellent antioxidant defense mechanisms thus ensuring better survival. In conclusion, Grade II and III glioma cells demonstrated coding region mtDNA mutations, leading to mitochondrial dysfunction and higher oxidative stress.

## Introduction

Glioma is the most common primary brain tumor and is well-known for the immense challenges in terms of management and understanding its pathobiology (Ordys et al., 2010; Walker et al., 2011). Despite therapeutic advances and recent discovery of molecular signatures from integrative genomic analysis, the patients' outcome remained poor. Gliomas are a group of heterogeneous tumors most widely classified using the revised WHO grading system. This classification scheme mainly focuses on cellularity, mitotic activity, nuclear atypia, vascularity, and necrosis (Walker et al., 2011), which are mostly of cell cycle and angiogenesis related pathways. Despite tremendous progress in discovering nuclear oncogenes and tumor suppressor genes, there are still many aspects of tumorigenesis that cannot be explained, such as predicting tumor behavior, aggressiveness, and risk of recurrence (Lu et al., 2009). Since nuclear causes did not fully explain the mechanisms of tumorigenesis, studying the tumor mitochondria opens a window of opportunity as it plays a central role in energy production, oxidative phosphorylation (OXPHOS), and apoptosis (Lueth et al., 2009; Verschoor et al., 2013).

Mitochondria are the only extra-chromosomal organelle which carries DNA (Kulawiec et al., 2010). Previously, the field of mitochondria research was neglected but recently regained attention due to its proposed ability to dysregulate cancer cell energenetics, influence biosynthetic pathways and controlling the oxidative stress status, thus leading to tumor progression (Wallace, 2012). Mitochondria have relatively small, compact and unique DNA consisting of 16,569 base pairs, coding for 13 respiratory complex proteins, 22tRNAs, and 2 rRNAs (Wallace, 2012). It is unique for the presence of multiple sets of DNA per mitochondria, multiple copies per cell and therefore mutations could accumulate partially leading to a heteroplasmy state. Its small size, abundance in number and well-protected circular form are advantageous for targeting it as screening tools for limited clinical samples such as glioma. It is well-known that most solid tumors, including gliomas, possess abnormal cellular metabolism (Ordys et al., 2010). Mitochondrial DNA mutations, as well as certain nuclear encoded mutations, could lead to possible respiratory chain and OXPHOS dysfunction. The altered cellular respiratory system may generate excessive reactive oxygen species (Petros et al., 2005), enhance tumorigenesis and further lead to a vicious cycle of DNA damages (Lu et al., 2009; Kulawiec et al., 2010; Wallace, 2012). mtDNA is also involved in regulation of cellular apoptosis which is an important target for cancer biology. Therefore, mitochondrial DNA mutations could possibly be the key regulators in cancer initiation, maintenance and progression.

Mitochondrial DNA mutations have long been documented in various tumors including gliomas (Carew and Huang, 2002; Chatterjee et al., 2006; Lueth et al., 2009; Ordys et al., 2010; Larman et al., 2012). For example, Chattopadyay and colleagues identified 271 SNPs (7 novel and 15 somatic mutations) in mtDNA of 8 oral cancer tissues compared to reference (rCRS) and also their adjacent normal tissues (Chattopadyay et al., 2016). Vidone and colleagues characterized the mtDNA mutations in glioblastoma multiforme patients via The Cancer Genome Atlas Data Portal (TCGA, https://cancergenome.nih.gov/). They analyzed 21 whole exome sequencing (WES) and 28 whole genome sequencing (WGS) using the Binary Alignment/Map files from 45matched tumor-blood samples of GBM patients. In total, 1,218 mutations were identified in the tumor samples and were classified as tumor-specific mutations. About 1,193 (98.5%) of the tumor-specific mutations showed a heteroplasmy level ranging from 0.001 to 0.082 (Vidone et al., 2015). However, a significant number of these studies did not cover the whole mitochondrial genome but mainly focused on the non-coding D-loop region (Liu et al., 2001; Kirches et al., 2002; Nomoto et al., 2002; Aikhionbare et al., 2007). In addition, the relationship between mitochondria functions and oxidative stress status has not been explored and remained a challenging task (Carew and Huang, 2002). In this study, we determined the mitochondrial functions and oxidative stress status in different grades of glioma cell lines. We then employed a high throughput whole mitochondrial genome resequencing approach to sequence the whole mtDNA of these cell lines in order to detect mitochondrial DNA mutations which could possibly account for mitochondrial dysfunctions and oxidative stress in these cells.

## Materials and methods

### Cell culture

Three glioma cell lines, namely, LN18 (CRL–2610™), SW1783 (HTB–13™), and 1321N1, were obtained and maintained *in-vitro*. LN18 cells were purchased from ATCC® (Rockville, MD, USA) and cultured in DMEM with 5% fetal bovine serum (FBS) at 37°C humidified atmosphere containing 5% CO_2_. It was originally established in 1976 from aright temporal lobe tumor of a 65-year-old Caucasian man with a Grade IV poorly differentiated glioblastoma (Diserens et al., 1981). The SW1783 cell line from ATCC, representing Grade III anaplastic astrocytoma from a 68-year-old Caucasian man, was maintained in Leibovitz's L-15 medium with 10% FBS at 37°C humidified atmosphere of 100% air. The 1321N1 cells, a grade II human brain astrocytoma cell line acquired from ECACC (Salisbury, Wiltshire, UK), was cultured in DMEM with 10% FBS at 37°C humidified atmosphere containing 5% CO_2_. The 1321N1 cell line was isolated in 1972 as a sub clone of 1181N1 cell line which was derived from the parent line U-118 MG by Potten and Macintyre (1968). All cell culture grade reagents were obtained from GIBCO (Life Technologies, Gaithersburg, MD, USA) and Sigma (St Louis, MO, USA). All cell culture-ware products were supplied by Nunc (Rochester, NY, USA). A commercially available primary culture of normal human astrocyte (NHA) from LONZA (Walkersville, MD, USA) that was isolated from a 19 weeks male human fetus was also propagated to serve as the normal control. NHA cells were cultured using the manufacturer's recommended media (AGM™ BulletKit™, LONZA) at 37°C humidified atmosphere containing 5% CO_2_. For all the parameters listed below, 3 passages of each cell line were harvested in different days and the experiments were performed on the same day of cell harvesting. (For LN-18 & SW1783, cells from passages 4 to 10 after resuscitation were used; for 1321N1, cells from passages 5 to 12 were used, whereas for NHA, cells between passages 4 and 7 were used). All experiments were performed in technical triplicates and in 3 wells for each passage of cell line. Cell viability above 90% was confirmed in all experiments using 0.25% Trypan blue staining.

### Whole mitochondrial genome sequencing

Genomic DNA of each cell line was extracted using the QIAamp DNA Mini Kit (Qiagen, Hilden, Germany) according to manufacturer's instructions. The DNA was then subjected to high throughput whole mitochondrial genome sequencing using the GeneChip Human Mitochondrial Resequencing Array 2.0 (Affymetrix, Santa Clara, CA, USA; Maitra et al., 2004; Sui et al., 2006; Zhou et al., 2006; Dasgupta et al., 2009). This chip is an oligonucleotide tiling array for mtDNA resequencing. It interrogates the entire 16 kb mitochondrial genome with oligonucleotide probes synthesized *in situ* using standard resequencing array tiling strategy of eight unique 25-mer probes per base position and 4 oligonucleotide probes per strand. Three overlapping long range PCR were performed to amplify the whole mitochondrial genome from 75 ng of total genomic DNA using high fidelity LA PCR kits v2.1 (TaKaRa Bio Inc, Madison, WI, USA) and 3 pairs of array manufacturer suggested primers (Mito1, Mito2, and Mito3). A positive PCR control using the CustomSeq kit included IQ-EX Control Template and 7.5 kb primer pair was carried out concomitantly (Affymetrix, Santa Clara, CA, USA). The desired bands of PCR products were excised from the agarose gel under direct visualization using PrepOne Sapphire blue LED light illuminator (Embi Tec, SanDiego, CA, USA) after SYBR® Safe (Invitrogen, Gaithersburg, MD, USA) gel staining. Gel pieces were then purified with QIAquick Gel extraction kit (Qiagen, Hilden, Germany). Gel electrophoresis to reconfirm the targeted PCR bands was performed prior to pooling, fragmentation, labeling and hybridization. All subsequent steps were performed following the standard GeneChip CustomSeq Resequencing Array Protocol. Hybridized chips were washed using GeneChip Fluidics Station 450 and scanned with GeneChip Scanner 3000 (Affymetrix, Santa Clara, CA, USA). Generated data was then analyzed using GeneChip Sequence Analysis (Affymetrix, Santa Clara, CA, USA) software with algorithm analysis parameters set at genome model = diploid to detect heteroplasmy changes and quality score threshold (QST) = 3 for optimal overall base calling and call accuracy. The resulting sequences were compared to the revised Cambridge Reference Sequence (rCRS, GenBank # NC_012920) and sequence alterations were confirmed only if the same changes occurred in all triplicate results. The heteroplasmy percentage and ratio of expected alleles (REA) were calculated using the array probe intensity information according to Coon's method (Coon et al., 2006).

### Phylogenetic analysis and protein pathogenicity prediction

The mtDNA sequence variations of the cell lines were then subjected to further analysis using the Phylotree.org (http://www.phylotree.org) which is a comprehensive phylogenetic tree of global human mitochondrial DNA variation (van Oven and Kayser, 2009). Haplogrep (http://haplogrep.uibk.ac.at/), a fast and reliable bioinformatics algorithm for automatic classification of mtDNA haplogroups (Kloss-Brandstätter et al., 2011) was also employed to build the phylogenetic tree of each sample according to global human mitochondrial DNA haplogroup nomenclature and thus, obtain a true disease-related mitochondrial mutation. Apart from being able to filter out haplogroup specific variant of mtDNA differences, this also serves as a quality control step to detect sample cross-contamination and technical artifacts (Salas et al., 2005). The filtered results likely represent true disease-related sequence changes and were examined against MITOMAP database (http://mitomap.org/MITOMAP).

Polymorphism Phenotyping v2 (PolyPhen2, http://genetics.bwh.harvard.edu/pph2/) which uses a multiple sequence alignment pipeline together with structure based features was used to determine the impact of amino acid changes on the function and structure of downstream proteins via physical and comparative considerations (Adzhubei et al., 2010). The generated Protein Function Prediction Score (HumDiv) was scaled into either benign, possibly damaging or probably damaging. We also used the pMut (http://mmb2.pcb.ub.es:8080/PMut/), a web based server that could retrieve information from database of mutation hotspots, utilizes neural network to analyze the single nucleotide polymorphisms (SNPs) and thus annotate the pathological character of single point amino acid mutations (Ferrer-Costa et al., 2004).

### Validation of the mtDNA variations using sanger sequencing and TaqMan mutation detection assay

Samples with non-synonymous coding region mutations identified by the GeneChip Human Mitochondrial Resequencing Array were further validated using conventional dye terminator sequencer. In total, 3 pairs of primers were designed accordingly using NCBI primer blast to amplify each specific mutational locus. The PCR products were resolved using agarose gel electrophoresis and purified with QIAquick PCR purification kit (Qiagen, Hilden, Germany). Cycle sequencing was performed using the Big Dye Terminator V3.1 reagent (Life Technologies, Gaithersburg, MD, USA). The cycle sequencing products were then sequenced using the ABI 3130XLgenetic analyzer (Life Technologies, Gaithersburg, MD, USA). The results were analyzed with the ABI Sequencing Analysis Software v5.1 (Life Technologies, Gaithersburg, MD, USA) and matched using NCBI Basic Local Alignment System Tool (BLAST) and CLC Sequence Viewer 6 (CLC Bio, Denmark). In order to identify the low level of heteroplasmy which could not be detected by conventional dye terminator sequencing method, the Taqman SNP genotyping assay (Life technologies, Gaithersburg, MD, USA) was performed. The locus of interest was amplified using custom designed PCR primers and probe set. Real time quantitative PCR was performed and readings were analyzed using allelic discrimination analysis software to identify the percentage of heteroplasmy of each allele.

### Oxidative stress measurement using CM-H_2_DCFDA dye

Cells were seeded into sterile, tissue culture treated u-Slide 8 Well chamberslides (Ibidi, GmBH, Germany) according to its usual seeding density and grown to 50–70% confluency. To estimate the intracellular generation of ROS, cells were washed with warm 1xPBS twice before being labeled with 5 μM CM-H_2_DCFDA (Molecular Probes, Invitrogen, CA, USA), 300 nM DAPI nucleic acid stain and incubated for 45 min at 37°C in its standard culture condition. Then each u-slide was transferred to the confocal microscopic station with 37°C heated chamber supplied with 5% CO_2_ for live cell imaging. Laser intensity, gain and offset were standardized for all cell lines and each passage. Tile scan images were captured randomly using the TCS-SP2 confocal microscopy system (Leica Microsystem, Nussloch GmbH, Germany). CM-H_2_DCFDA is a general oxidative stress indicator that exhibits long term fluorescent retention in live cells. The intensities of CM-H_2_DCFDA (Excitation/Emission: 495/520 nm), which reflects the amount of intracellular ROS, were measured from three passages of each cell line that were seeded in three wells every passage. For each cell line, a total of 20 different microscopic fields per passage that contained comparable number of cells were integrated using LAS AF image analysis software (Leica Microsystem, Nussloch GmbH, Germany) to determine its mean pixel intensity value.

### Mitochondrial mass and mitochondrial oxidative activity detection using Mitotracker Green (MTG) and Mitotracker Orange (MTO)

For relative mitochondrial function, stains for MTG and MTO from Invitrogen Molecular Probes (Invitrogen, CA, USA) were utilized. MTG in the mitochondrial lipid environment will fluoresce regardless of the membrane potential and serves as the dye for mitochondrial physical mass quantification. On the other hand, MTO will only be fluorescent once it is taken into live cells and converted by actively respiring mitochondria into its fluorescent state, allowing estimation of oxidative activity. Therefore, by combining both stains, the relative mitochondrial function with its mitochondrial mass as baseline can be obtained (Morici et al., 2007; Agnello et al., 2008). Similarly, cells were seeded into 8-well chamber slide and grown into 50–70% confluency. After washing 2x with warm 1xPBS, cells were stained with 100 uM MTG and 500 uM MTO in DPBS+Ca/Mg/Glucose to maintain its normal metabolic state. Cells were then incubated for 45 min at 37°C in its standard culture condition. The u-slide was then transferred to the same confocal microscopic station with 37°C heated chamber supplied with 5% CO_2_ for live cell imaging. Laser intensity, gain and offset were standardized for all cell lines and each passage. Tile scan images were captured randomly using the same TCS-SP2 confocal microscopy system (Leica Microsystem, Nussloch GmbH, Germany) with sequential detection for both stains. The intensities of MTO (Excitation/Emission: 554/576 nm) and MTG (Excitation/Emission: 490/516 nm) were measured from four passages of each cell line that were seeded in three wells every passage. For each cell line, a total of 20 different microscopic fields per passage that contained comparable number of cells were integrated to analyze each fluorescence mean pixel intensity value. The ratio of MTO intensity: MTG intensity was then calculated and named as mean index of MTO: MTG which reflects its relative mitochondrial function.

### Total antioxidant capacity assay

The antioxidant capacity of the glioma and NHA cells was quantitatively measured using the Antioxidant Assay Kit (Sigma, St. Louis, MO, USA). It utilizes the ferryl myoglobin radical formation from metmyoglobin and hydrogen peroxide to produce the radical cation of ABTS (2-2′-azino-bis 3-ethylbenthiazoline-6-sulfonic acid) which is a soluble green chromogen that could be spectrophotometrically determined at 405 nm. This assay also used a water soluble vitamin E analog, Trolox, as the standard control for antioxidant. Approximately 0.5 × 10^6^ cells were trypsinized, washed twice in 1xPBS and resuspended in 250 ul 1x kit assay buffer and sonicated on ice for 1.5 min. Cell lysates were then centrifuged at 12,000 × g for 15 min and the supernatant was collected on ice for further assay procedures. The following steps were performed according to the standard kit protocol and final solutions were read at 405 nm on a 96 well microplate reader (Biotek, WI, USA). Results were calibrated using the reference curve generated from the Trolox standard control. For final protein normalization, the corresponding protein concentration of each cell lysate was determined using Quick-start Bradford 1x Dye Reagent with Bovine Serum Albumin as standard control (Bio-Rad Lab., CA, USA). After 5 min incubation on orbital shaker, the lysate and reagent mix were read at 595 nm on 96 well microplate reader to measure the protein content (Biotek, WA, USA). All the reference curves were accepted only if *r*^2^ > 0.992 and the final result of each cell lines was obtained in technical triplicates. The results for antioxidant capacity were then divided by the corresponding protein concentration and the mean antioxidant concentration was expressed in relative to mmol Trolox standard/g protein.

### DNA oxidation detection using 8-OHdG

The 8-OHdG level which represents the DNA oxidation marker was measured using the enzyme immunoassay (EIA) that utilizes anti-mouse IgG coated plate and a tracer consisting of an 8-OHdG-enzyme conjugate. In total, 1 × 10^7^ cells of each cell line were harvested and washed with 1xPBS twice before extracting the DNA using the sodium iodide method, which is a less oxidizing method, according to manufacturer's instruction (Wako DNA extractor TIS kit, Wako, Japan). Quality and quantity of the DNA samples were measured using NanoDrop. All samples were then prepared for enzymatic digestion of DNA using the Wako 8-OHdG preparation kit (Wako, Japan). A total of 20 ug DNA of each sample and 5 ug for NHA was aliquoted and diluted to 150 ul in Milli-Q water. The end products were filtered using Vivaspin 500 (Sartorius, Germany) and kept on ice. These samples were then subjected to 8-OHdG EIA (Cayman, USA) in technical triplicates on the same day. The cold DNA filtrates were then diluted eight-fold using the EIA buffer (NHA-diluted two-fold) which made the final DNA concentration in each sample 5 ng/ul. The standard steps of EIA kit protocol were performed accordingly. The final solution was incubated overnight in 4°C, washed, added in tracer, incubated again in dark and read at 405 nm at 30 min interval. The final 8-OHdG measurement result was obtained in pg/ug DNA.

### Statistical analysis

For all the above biochemical assays, the data were analyzed using one-way ANOVA with test for homogeneity of variance and Welch correction (IBM SPSS ver 20 software). Subsequently, comparison between sample groups was further performed using either Games-Howell *post hoc* test or Tukey HSD test depending on the result of homogeneity of variance. Significant level of difference was taken at *p* < 0.05.

## Results

### Mtdna sequence variations in glioma and normal human astrocyte cell lines

mtDNA was successfully amplified using long range PCR. The PCR products were subjected to whole mitochondrial genome resequencing array using thePCR IQ-EX template as the quality control reference. All the generated results were analyzed using Gseq software and the average call rates (percentage of each sequence were assigned a positive nucleotide call) were all above 95.8%. These call rates exceeded the 94.6% call rate achieved by the chip developer team in year 2006 (Zhou et al., 2006). After comparison with rCRS, a total of 16.33–32.00 sequence variations were detected on average for each cell line. The average sequence variations were narrowed down to 15–31 after considering mutations detected in all 3 passages of each cell line (Table 1).

**Table 1:** **Summary of GeneChip Human Mitochondrial Resequencing Array 2.0 results**.

**Cell Line**	**NHA**	**1321N1**	**SW1783**	**LN18**
Type	Normal Human Astrocyte	Astrocytoma	Anaplastic Astrocytoma	Glioblastoma Multiforme
Grade	–	II	III	IV
Source	LONZA	ECACC	ATCC	ATCC
Average Resequencing Array Call Rate (total sequence analyzed = 16,544 bp/chip; triplicates each cell line)	96.1%	96.0%	95.8%	96.0%
Average sequence variations (in comparison to rCRS)	17.67	16.33	32.00	27.33
Confirmed sequence changes	16	15	31	24
Haplogroup assignment	B2	U5b2c2	H13a1a1a	H1e1b1
Haplogroup match	78.5%	94.3%	96.1%	96.9%
Total sequence variations after assigning phylogenetical haplogroup	5	6	2	5
Novel mutations	0	3	0	2
D-Loop involvement (%)	2 (40.0%)	0 (0%)	0 (0%)	1 (20.0%)
Coding region involvement (%)	0 (0%)	4 (66.7%)	2 (100.0%)	3 (60.0%)
tRNA involvement (%)	2 (40.0%)	2 (33.3%)	0 (0%)	0 (0%)
Amino acid changes (%)	0 (0%)	2 (33.3%)	1 (50.0%)	0 (0%)
Amino acid / silent change ratio	0	1.0	1.0	0
A>G transitions (%)	1 (20.0%)	3 (50.0%)	1 (50.0%)	1 (20.0%)
Heteroplasmy (%)	1 (20.0%)	6 (100.0%)	0 (0%)	3 (60.0%)

**Figure 1:**
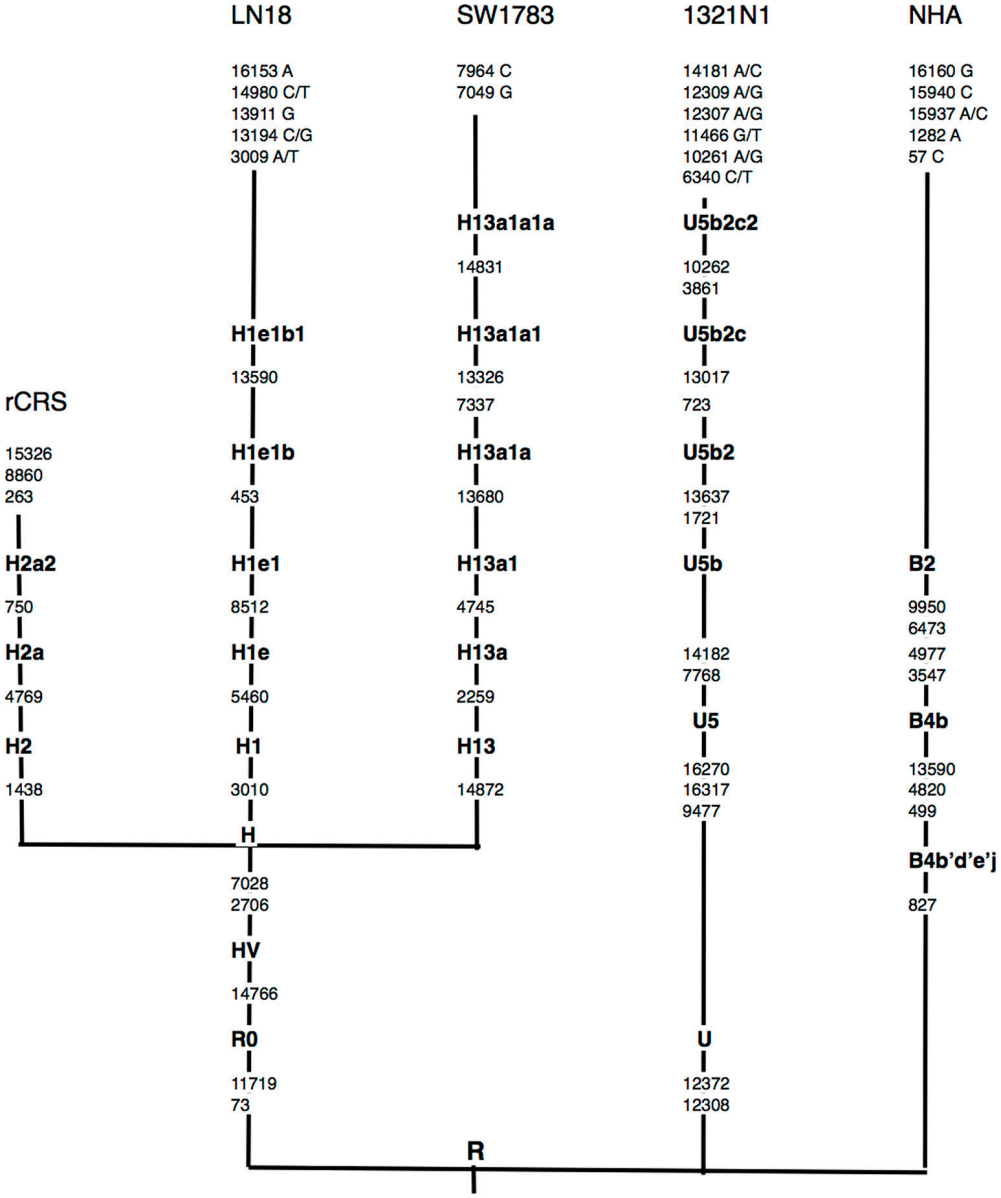
**Schematic representation of mtDNA sequence phylogenetic tree**. mtDNA sequence phylogenetic tree constructed from all 3 different grades of glioma, normal human astrocyte (NHA) cell lines and the revised Cambridge reference sequence (rCRS) [NC_012920]. Suffixes indicate transitions, “/” indicates heteroplasmy changes. Word in bold along the lineage line represent haplogroup names. Nucleotide changes after the most distal haplogroup assignment were considered as non-lineage specific, likely somatic sequence alterations.

Using the Phylotree.org and HaploGrep for phylogenetic tree reconstruction, we were able to show that all samples followed a distinct phylogenetical haplogroup nomenclature of European or Caucasian lineage and there was no sample cross-contamination between cell lines or laboratory personnel (Figure 1). This additional step also provided us the advantage to filter out the haplogroup related polymorphisms since there was neither matched leucocyte nor normal tissue sample available to be our germline reference. Thus, we assigned those sequence variations that remained after phylogenetical haplogroup filtering as likely somatic mutations.

In total, 2–6 of sequence changes were detected in all cell lines. Of note, 3 sequence changes from Grade II and 2 sequence changes in Grade IV glioma cell line were novel mutations but NHA did not harbor any novel mutation. Another important finding was that almost 60% of the mutations in all cell lines were located at the coding region. The NHA cell line showed no mtDNA mutation in the coding region. The LN18, Grade IV glioblastoma cell line, showed only synonymous mutations where none of the sequence changes led to changes in the amino acid sequences. TheSW1783 and 1321N1 cell lines showed 1 and 2 non-synonymous mutation respectively. Half of the sequence changes in 1321N1 and SW1783 were of A > G transitions which were known as a consequence of oxidative stress induced DNA damage. All of 1321N1 mtDNA mutations were heteroplasmy but all SW1783 changes were homoplasmy.

All the nucleotide changes in NHA were at non-coding regions and were polymorphisms which have been reported elsewhere (Table 2). In glioma cell lines, the coding region changes were located at *ND3, ND4, ND5, ND6, CO1, CO2*, and *CYTB* genes. The 3 mutation spots for the 1321N1 and SW1783 cells were located at *ND6, CO1* and *CO2* respectively. 1321N1 harbored a C6340C/T mutation which was previously reported as a somatic mutation in prostate cancer. This 41.5% heteroplasmy sequence change led to threonine to isoleucine production switch at the *CO1* locus in which pMUT prediction showed as pathological with a reliability index of 9 but PolyPhen2 showed a prediction score of 0.025, hence a benign mutation (Table 3). Another non-synonymous mutation was A14181A/C in the *ND6* gene with a 18.3% heteroplasmy change leading to changes in amino acid tyrosine to aspartate. Both pMUT and PolyPhen2 predicted this SNP as pathological and probably a damaging protein change. On another hand, T7064C which occurred in the SW1783 cells was a homoplasmy mutation in locus *CO2* causing phenylalanine to leucine change. This mutation similarly was predicted to be pathological and probably damaging.

**Table 2:** **mtDNA sequence variations in glioma and normal human astrocyte cell lines**.

**Cell Line**	**Nucleotide Position**	**Nucleotide Change**	**Hetero-plasmy**	**Gene Locus**	**RNA**	**Amino Acid Change**	**Remarks**
NHA	57 1282 15937 15940 16160	T > C G > A A > A/C T > C A > G	No No Yes No No	MT-DLOOP MT-RNR1 MT-TT MT-TT MT-DLOOP	– 12s rRNA tRNA Thr tRNAThr –	Non coding Non coding Non coding Non coding Non coding	Reported polymorphism Reported polymorphism Reported polymorphism Reported polymorphism Reported polymorphism
1321N1	6340	C > C/T	Yes	MT-CO1	−	Thr to Ile	Reported polymorphism
							& somatic mutation in
							Prostate cancer
	10261	A > A/G	Yes	MT-ND3	−	Synonymous	Novel changes
	11466	T > G/T	Yes	MT-ND4	−	Synonymous	Novel changes
	12307	A > A/G	Yes	MT-TL2	tRNALeu	Non coding	Reported polymorphism
	12309	A > A/G	Yes	MT-TL2	tRNALeu	Non coding	Reported polymorphism
	14181	A > A/C	Yes	MT-ND6	−	Tyr to Asp	Novel changes
SW1783	7049 7964	A > G T > C	No No	MT-CO1 MT-CO2	– –	Synonymous Phe to Leu	Reported polymorphism Reported polymorphism
LN18	3009	C > A/T	Yes	MT-RNR2	16s rRNA	Non coding	Novel changes
	13194	G > C/G	Yes		−		
	13911	A > G	No	MT-ND5	−	Synonymous	Novel changes
	14980	C > C/T	Yes	MT-ND5		Synonymous	Reported polymorphism
	16153	G > A	No		−		
				MT-CYB		Synonymous	Reported polymorphism
				MT-DLOOP		Non coding	Reported polymorphism

*Sequence changes found in all 3 different grades of glioma cell lines and a normal human astrocyte cell line after filtering out phylogenetically related polymorphism sites. All sequence changes were labeled as novel changes if no previous reports could be retrieved from MITOMAP database (last edited 22 May 2016: MITOMAP: Reported Mitochondrial DNA Base Substitution Diseases rRNA/tRNA Mutations. 20 May 2016: MITOMAP: Reported Mitochondrial DNA Base Substitution Diseases: Coding and Control Region)*.

*List of abbreviations: MT-DLOOP (regulatory region of the mtDNA), MT-RNR1 (mitochondrially encoded 12S RNA gene), MT-TT (mitochondrially encoded tRNA Threonine gene), MT-CO1 (Mitochondrial Cytochrome Oxidase Subunit 1 gene), MT-ND3 (Mitochondrial NADH Dehydrogenase 3 gene), MT-ND4 (Mitochondrial NADH Dehydrogenase 4 gene), MT-TL2 (Mitochondrially encoded tRNA Leucine 2 gene), MT-ND6 (Mitochondrial NADH Dehydrogenase 6 gene), MT-CO2 (Mitochondrial Cytochrome Oxidase Subunit 2 gene), MT-RNR2 (mitochondrially encoded 12S RNA gene), MT-ND5 (Mitochondrial NADH Dehydrogenase 5 gene), MT-CYB(mitochondrially encoded Cytochrome B gene), rRNA (ribosomal ribonucleic acid), tRNA (transfer ribonucleic acid), Thr (Threonine), Leu (Leucine), Ile (Isoleucine), Tyr (Tyrosine), Asp (Asparagine), Phe (Phenylalanine)*.

**Table 3:** **Predicted functional significance of protein alterations**.

**Cell Line**	**Nucleotide Change**	**Hetero-plasmy; percent-age (%); REA**	**Gene Locus**	**Amino Acid Change**	**PolyPhen-2 Protein Function Prediction Score (HumDiv); Specificity/Sensitivity**	**Pmut Prediction (Score); Reliability score**	**Protein Encoded; Respiratory Complex Involvement & Related Functions**
1321N1	C6340C/T	Yes; 41.5%; REA 0.29	MT-CO1	Thr to Ile	0.025 Benign; 0.81/0.95	0.9582 Pathological; reliability 9	Cytochrome C Oxidase Subunit 1 (Complex IV) - Functional core of enzyme complex that catalyzes the reduction of oxygen to water in respiratory chain. Subunit 1 is the catalytic subunit.
	A14181A/C	Yes; 18.3%; REA 0.60	MT-ND6	Tyr to Asp	0.940 Probably damaging; 0.94/0.80	0.9263 Pathological; reliability 8	NADH-ubiquinone Oxidoreductase Chain 6 (Complex I) - Core subunit of the mitochondrial membrane respiratory chain NADH dehydrogenase (Complex I) that is required for catalysis and transfer of electrons from NADH to the respiratory chain
SW1783	T7964C	No	MT-CO2	Phe to Leu	0.993 Probably damaging; 0.97/0.70	0.7318 Pathological; reliability 4	Cytochrome C Oxidase Subunit 2 (Complex IV) - Functional core of enzyme complex that catalyzes the reduction of oxygen to water in respiratory chain. Subunit 2 transfers the electrons from cytochrome c to the bimetallic center of the subunit 1

*Bioinformatics prediction of protein pathogenicity from amino acid change in mitochondrial DNA missense mutation detected from 1321N1 and SW1783 cell lines. Heteroplasmy percentage and ratio of expected allele (REA) was calculated using Coon's method*.

*List of abbreviations: MT-CO1, Mitochondrial Cytochrome Oxidase Subunit 1 gene; MT-CO2, Mitochondrial Cytochrome Oxidase Subunit 1 gene; MT-ND6, Mitochondrial NADH Dehydrogenase 6 gene*.

**Figure 2:**
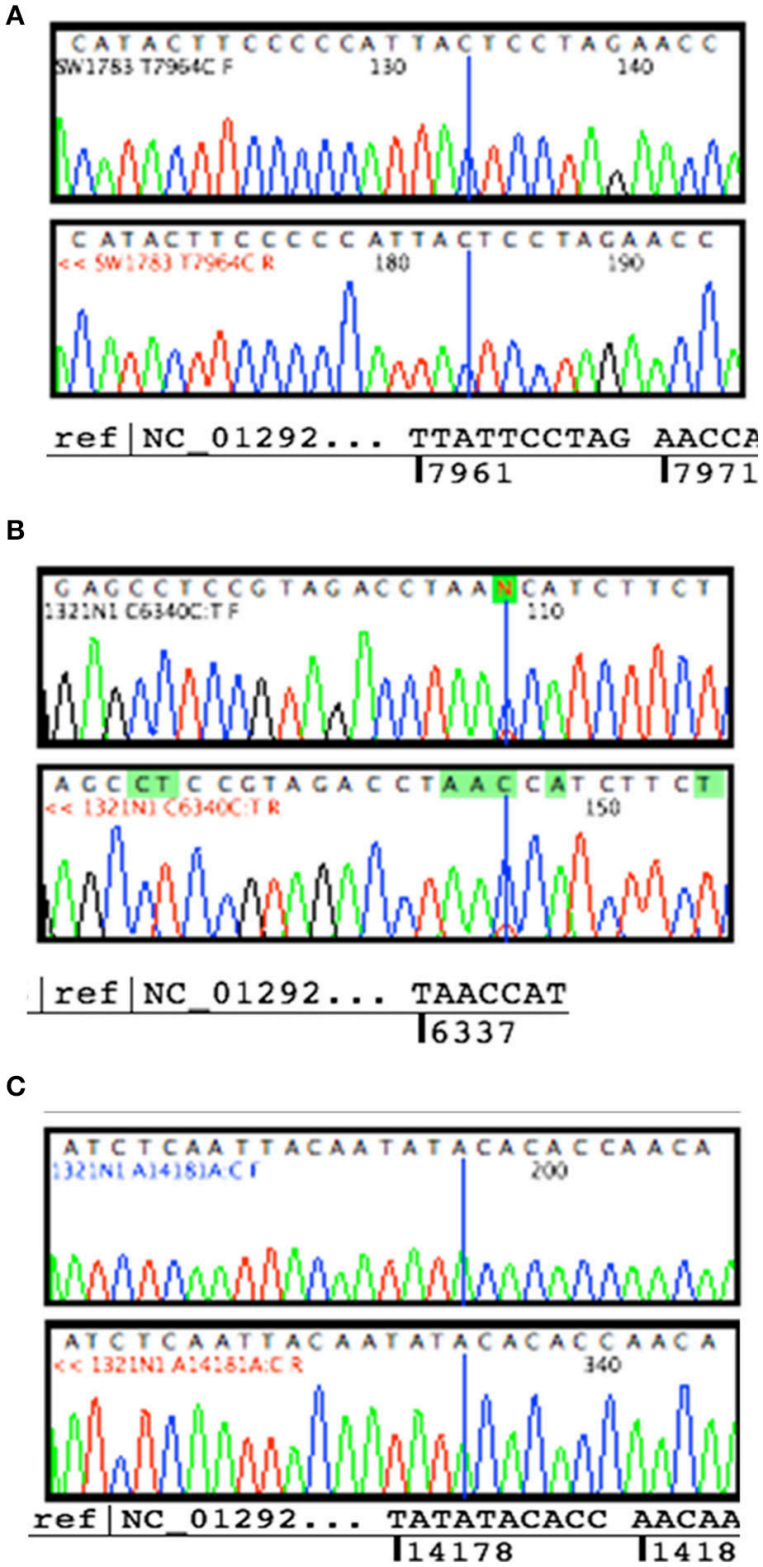
**Validation for non-synonymous mutations. (A)** Homoplasmy change of non-synonymous mutation (T7964C) found in SW1783 cells were confirmed. **(B)** Detection of high level heteroplasmy C6340C/T (41.5%) using Sanger sequencing method. **(C)** Confirmation of low level heteroplasmy of A14181A/C (18.3%) in 1321N1 using Taqman probe SNP Genotyping Assay.

Sanger sequencing successfully confirmed the homoplasmy mutation of SW1783 and C6340C/T high level heteroplasmy change in 1321N1 (Figures 2A,B). However, it was not able to validate the low level heteroplasmy mutations (Figure 2C). A more sensitive Taqman probe based SNP Genotyping assay was then used to overcome the limitation of low detection level for heteroplasmy using Sanger sequencing.

### Mitochondrial functions in glioma and normal human astrocyte cell lines

**Figure 3:**
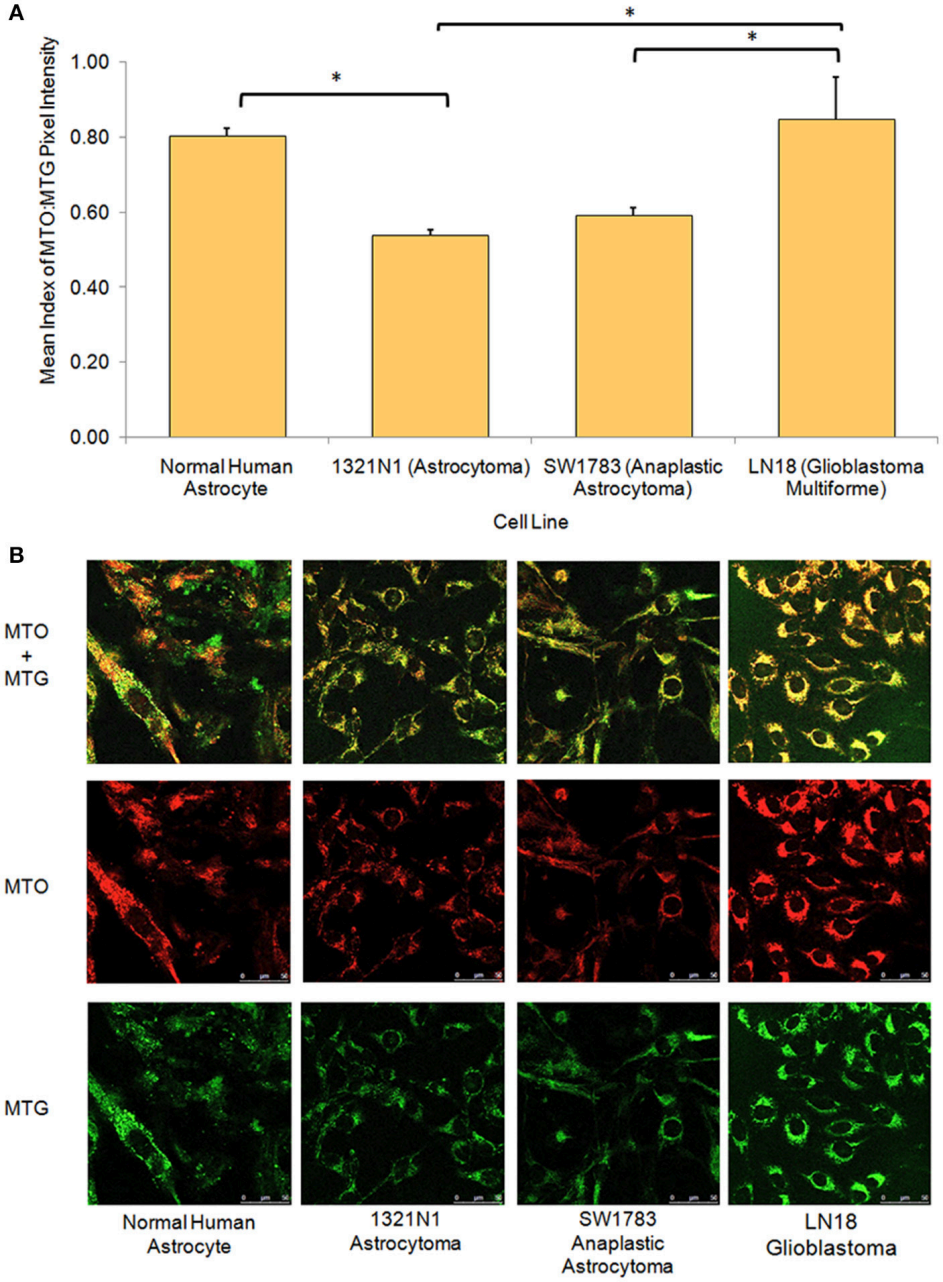
**Relative mitochondrial functions in different grades of glioma cell lines and normal human astrocyte. (A)** Bar chart showing significantly lower level of mitochondrial function:mass ratio in grade II 1321N1 and grade III SW1783 cell line (^*^indicates *p* < 0.05 using one-way ANOVA, bars represent mean MTO:MTG pixel intensity index ± 1 SEM, mean index value as shown in bars). **(B)** Relative mitochondrial functions were demonstrated using MitoTracker Orange (MTO) stain where relative mitochondrial mass was shown by MitoTracker Green (MTG) stain. Each stain was captured using CLSM with sequential detection. Images merged and analyzed quantitatively using LAS AF software to generate the relative function ratio.

From the live cell confocal laser scanning microscopic evaluation of relative mitochondria function, we were able to show that the 1321N1 and SW1783 cell lines which harbored non-synonymous mtDNA mutations had significantly poor mitochondrial functions compared to the LN18 and NHA cells (Figure 3). The simultaneous measurement of MTO and MTG stains allowed us to evaluate the mitochondrial mass and relative oxidative activity separately and integrate both for a better understanding of their relative mitochondrial functions. It was clear that the merged MTO & MTG stains of Grade II and III cells were more of yellow as the intensities of green and red were equivalent, whereas the other 2 cell lines verged toward red.

### Oxidative stress status in glioma and normal human astrocyte cell lines

**Figure 4:**
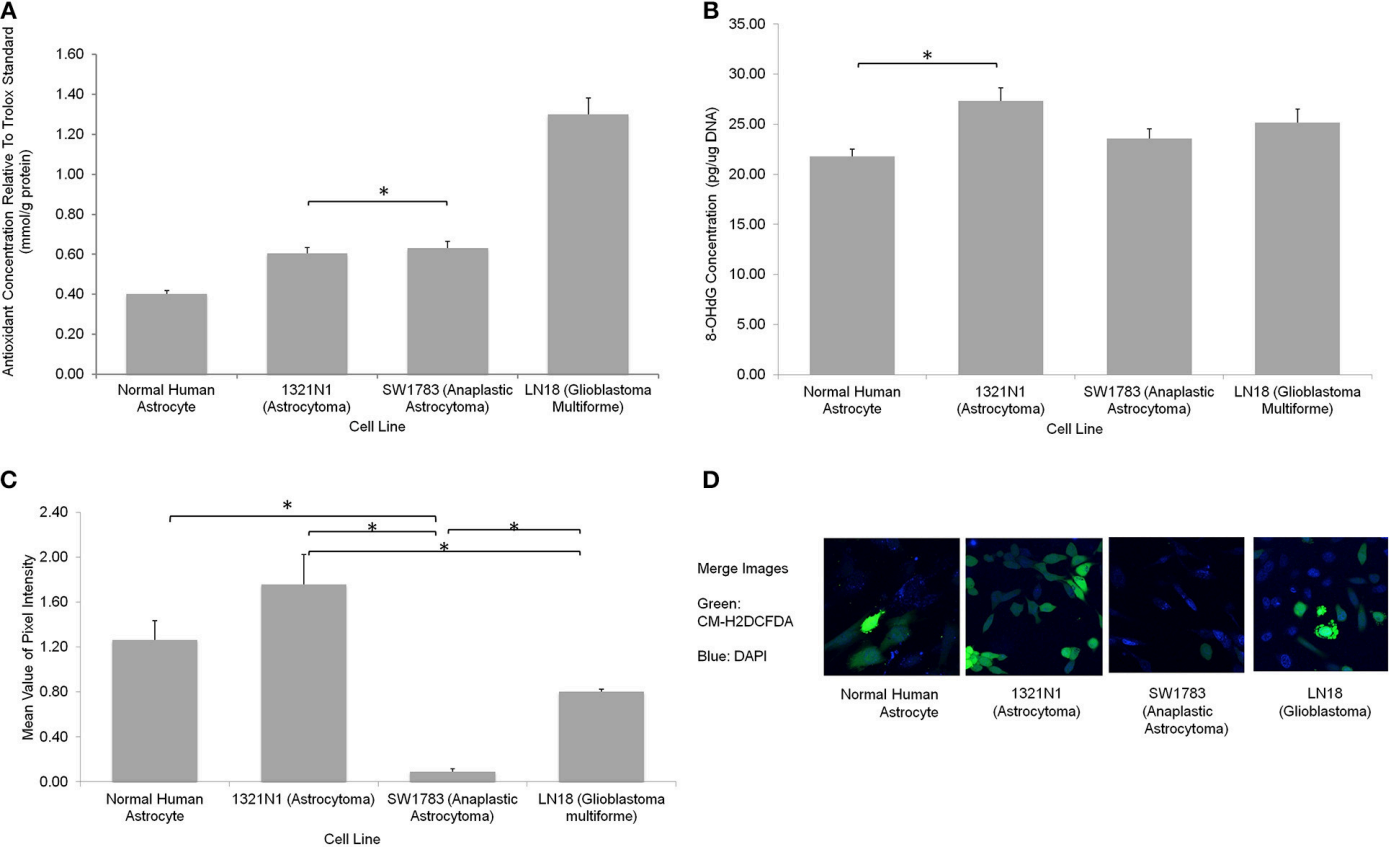
**Oxidative stress status in different grades of glioma cell lines. (A)** A significantly higher total antioxidant capacity demonstrated by LN18 glioblastoma multiforme cells (*p* < 0.05 using one-way ANOVA). **(B)** The DNA oxidation marker, 8-OHdG level was higher in grade II 1321N1 cells which was also harboring the most coding region non-synonymous mtDNA mutations (^*^indicates *p* < 0.05 using one-way ANOVA, bars represent mean 8-OHdG concentration ± 1 SEM, mean values as shown in bars). **(C)** Direct staining of CM-H_2_DCFDA in live cells showed that grade II 1321N1 cells sustained highest level of oxidative stress *in-vitro* (^*^indicates *p* < 0.05 using one-way ANOVA, bars represent mean pixel intensity ± 1 SEM, mean values as shown in bars). **(D)** Images of cells stained with CM-H_2_DCFDA and DAPI clearly demonstrated that 1321N1 cells harbored the highest amount of oxidative stress marker. As compared to the other cell lines, some cells detected by DAPI did not showed any green fluorescence.

We further evaluated the levels of oxidative stress in these cell lines using several assays. The CM-H_2_DCFDA stain which indicates direct ROS detection were observed to be taken up more in 1321N1 cells (Figure 4). Majority of cells showed marked green fluorescence as compared to other cell lines suggesting increased ROS in these cells. The 8-OHdG EIA assay, which detects oxidative DNA damage, reconfirmed the fact that Grade II 1321N1 cells harbored highest DNA oxidation. This is consistent with our sequencing results as up to 50% of sequence variations were observed in 1321N1 and A > G transition in mtDNA is a well-known alteration that could be due to harmful oxidative stress. The spectrophotometric total antioxidant assay result would give an overall estimation of the capability of cells to withstand oxidative stress. The results showed that normal human astrocytes, Grade II, III, or IV cells distinctly differed in their antioxidant capacity in which LN18 cells had the highest antioxidant capability and this capability reduced correspondingly to their aggressiveness in lower grade glioma cells and normal human astrocyte. This means the Grade IV cells are likely to have a better endogenous response toward harmful oxidative stress.

## Discussion

We successfully sequenced the whole mitochondrial genome in 3 human glioma cell lines and one normal human astrocytoma cell line. The Grade II 1321N1 and Grade III SW1783 cells both harbored significant amount of coding region sequence variations that led to amino acid changes in the encoded proteins. These sequence changes, when analyzed with *in-silico* protein function prediction software, were found to be mostly hazardous and pathological to their functional outcome. These cells also showed poorer mitochondrial function from the *in-vitro* live cell imaging analysis. mtDNA mutations involving the coding regions were known to cause respiratory chain dysfunction and mitochondrial defects (Carew and Huang, 2002; Lu et al., 2009; Park et al., 2009). However, it was shown that despite mitochondrial dysfunction and increased ROS, the tumor cells did not encounter cell death but instead showed accelerated tumor growth (Petros et al., 2005).

It is well recognized that mitochondrial dysfunction from mtDNA mutations could lead to electron transport chain (ETC) instability, leakage of electrons, and subsequently increased ROS production (Carew and Huang, 2002; Lueth et al., 2009; Trachootham et al., 2009). We showed this clearly in the live cell imaging of general oxidative stress indicator, CM-H2DCFDA stain, and 8-OHdG EIA in which the 1321N1 cells had significantly higher oxidative stress level. 1321N1 cells were found to have non-synonymous changes in nucleotides coding for both respiratory complex I and IV. On the other hand, SW1783 cells, which contained a pathogenic sequence change in the respiratory complex IV region, did not express high oxidative stress. Complex I, located in the inner membrane of mitochondria, hold the important function of catalyzing the transfer of electrons from nicotinamide adenine dinucleotide (NADH) to coenzyme Q (coQ) at the entrance step to redox process. Complex I and complex III are important in maintaining normal ROS level. Inhibition of complex I and III function could lead to high production of ROS (Abu-Amero et al., 2005) and it may further result in failure of the antioxidant defense system, causing lack of antioxidant response mechanism (Chatterjee et al., 2011). It was widely accepted that a low to moderate level of ROS from impaired ETC could actually promote cell proliferation and enhance tumorigenesis, rather than inhibiting it (Carew and Huang, 2002; Petros et al., 2005; Lueth et al., 2009; Trachootham et al., 2009; Seoane et al., 2011). Petros and colleagues also showed that by introducing a pathogenic mtDNA mutation into a prostate cell line, the resultant cybrid cells showed higher ROS level with remarkably increased *in-vivo* tumor volume (Petros et al., 2005). This further supports the hypothesis that mtDNA may play a role in tumorigenesis. The presence of mtDNA mutations in pre-neoplastic lesions, as found by Dasgupta et al. indicated the possible early involvement of these mutations in tumor progression (Dasgupta et al., 2009). Studies encompassing other spectrum of cancers also proposed the likelihood that mtDNA mutations confer a selective growth advantage early in oncogenesis (Abu-Amero et al., 2005; Larman et al., 2012).

With mtDNA mutation causing raised ROS level, we showed evidence of a higher oxidative DNA damage from the ROS insult, especially in Grade II 1321N1 cells. The higher amount of A > G transition also favored this finding. This A > G transition was found to be consistent with the known mutagenic outcome of DNA oxidative damage caused by ROS generated in mitochondria (Abu-Amero et al., 2005). The resultant DNA damage could lead to genetic instability and would further increase the number of mtDNA and nDNA mutations. Following that, the vicious cycle can effectively amplify the oxidative stress, which lead to redox environment alteration and subsequently promote tumor development (Carew and Huang, 2002; Salazar-Ramiro et al., 2016). Yeung and colleagues studied mtDNA mutations in 11 GBM cell lines which have been generated from GBM patients (Day et al., 2013; Yeung et al., 2014). The most susceptible gene mutation was *ND6* and *ND4* has highest mutations, where both encoded for complex I of the ETC. For the non-coding regions, variants with highest frequency was the mt-Dloop and also in the origin of light strand replication (Yeung et al., 2014). However, they did not mention the glioma grades of all the samples sequenced. Even though, we analyzed one cell lines per grade of glioma, we observed similar results where mt-Dloop and ND genes were frequently mutated in glioma cell lines. Mutations identified were different in both study. Yeung and colleagues also sequenced 13 normal brain tissue samples. Surprisingly, none of mutations identified in our study were present in these 13 normal brain tissue samples (Yeung et al., 2014). Nevertheless, this observation may need to be confirmed using several cell lines and also mtDNA sequencing could be performed in GBM patients with matched normal-tumor tissue samples. Similarly, none of themutations identified in our study were observed by Chattopadyay et al. (2016) where they performed next generation sequencing (NGS) in the tumor and adjacent normal tissues of oral cancer patients. However, most of these mutations occurred at the mt-Dloop region (Chattopadyay et al., 2016). Differences in the mutations identified could be due to population and disease specific.

It was not surprising that the NHA cells had neither novel nor coding region sequence change. It was surprising that the most malignant cell line, LN18, harbored plenty of sequence changes but none resulted in amino acid changes. Consequently, the cells retained normal mitochondrial function and it was further proven in our live cell imaging experiments. The cells also exhibited a powerful oxidative ability of functioning mitochondria as compared to others. In addition, Grade IV cells were also found to have the highest antioxidant ability, which may have explained for its advantage to minimize possible deleterious ROS by their antioxidant defense mechanism leading to better cellular survival.

The findings in the Grade IV cells in our study infer to a few possibilities to explaining their mtDNA status. First, these cells were known for its highly malignant feature and strong survival ability. It could have other more potent oncogenic drivers rather than just relying on mtDNA mutation to induce ROS for tumor proliferation (Chatterjee et al., 2011). Secondly, in order to survive in a highly competitive environment, cells that exhibit intact mitochondrial function could have been positively selected and retained (Vega et al., 2004). Thirdly, it could also be possible that these cells have acquired a unique mtDNA repair mechanism that most of the harmful sequence changes detected were repaired before it led to clonal expansion (Chatterjee et al., 2011). Last but not least, these tumor cells could possibly replicate so fast that evolution causes mtDNA mutations that were once damaging to be diluted out after it had gained other stronger tumor driver mechanism.

One of the unique features of mtDNA is the possible presence of a heteroplasmy state. Harboring heteroplasmy changes does not imply that it is less detrimental. Park et al showed that, with increasing proportion of mutant mtDNA in tumor cells, both respiratory function and ATP synthesis declined but tumor growth was enhanced (Park et al., 2009). The team postulated that for mtDNA mutations to play a role in tumorigenesis, the optimal condition is likely to be a heteroplasmic state. They also proposed that mtDNA mutations occurred early in tumorigenesis and it usually starts as heteroplasmy changes. Larman et al. stated that the assessment of heteroplasmy may offer insights into the specific time-point of tumor evolution. If the sample displayed significantly different proportion of heteroplasmy mutations, for example, in our 1321N1 cell lines harboring 41.5% C6340C/T and 18.3% A14181A/C, it may imply that these mutations either arose independently, or one mutation clonally expanded followed by second mutation in a distinct subset of tumor cells. Otherwise, it could also be due to subtle replicative differences of each mutant DNA resulting in different levels of heteroplasmy.

Park et al. also noted that homoplasmy mutations were found more in the late stage of tumor. Once the tumor had fully adapted to a glycolytic metabolism, mutant mtDNA causing severe mitochondrial defect will likely be selected against, and residual synonymous mutations left were usually not harmful to the cells (Ordys et al., 2010). In our study we observed that Grade II cells showed more heteroplasmy mutations and Grade III mutations were more of homoplasmy. However, the Grade IV cells revealed a mixture of both. Probably it is an indicator that these Grade IV cells were still active in proliferation and new mtDNA mutations were persistently generated as compared to Grade III where replication stabilized and tumorigenesis was more quiescent.

In this study, we clearly showed the efficacy of using this high throughput whole genome sequencing array for fast and reliable sequencing of mtDNA. In view of the wide distribution of mutations found on the mtDNA locus, sequencing of the entire genome, instead of only the non-coding D-Loop, is necessary and crucial (Sui et al., 2006; Dasgupta et al., 2009; Kulawiec et al., 2010). This array was found to have a relatively excellent call rate (up to 94.6%) with >99.99%base call reproducibility and a high validation specificity (Zhou et al., 2006). It also has a remarkably good sensitivity for heteroplasmy. We were able to detect heteroplasmy level as low as 18.3% in all the triplicate of results. This, however, was not producible using conventional capillary sequencing. However, it is worth taking note that this array is not designed for detection of insertions, deletions or frame shifts (Sui et al., 2006).

Antonio Salas et al. (2005) suggested that phylogenetic tree tracing and haplogroup assignment are crucial and unavoidable steps in mtDNA mutation studies. This mainly serves to filter out haplogroup related polymorphism and retain true disease-related mutations. Therefore, the previous studies could have falsely included a large number of haplogroup-related polymorphism (Salas et al., 2005) and were not able to focus on true disease-related mutations to look for their functional implications and contributions in carcinogenesis (Kulawiec et al., 2010; Bi et al., 2011). In our study, where live cells for simultaneous functional experiments were needed, and no matched normal blood or adjacent tissue was available, this step is even more critical.

Our study also had several unavoidable limitations. First, there was no appropriate controls such as matched normal controls. To resolve the lack of normal blood or adjacent normal tissue for control study, we employed a primary culture of NHA to serve as our normal control. Second, we used one cell lines for each glioma grades, hence it is difficult to draw definite conclusion for this study. The limited number of cell line for each grade may not reflect the true phenotype of glioma samples as gliomas are known to be heterogenous. We must agree that there is a need to demonstrate the reproducibility of mtDNA mutation patterns in tumor tissues of different grades before any firm conclusion can be drawn on findings related inherently to the grades of gliomas. The mtDNA sequencing should also be carried out in the glioma patients using matched normal and tumor tissue samples. Third, functional validation of the gene mutations were not included in this paper. Heteroplasmy condition is common for mtDNA diseases particularly in the tissues, as well as in the cell lines. The threshold of the mutant and wild type may be varies due to different passages in the culture, hence the functional aspects of these mutations is difficult to predict (Tuppen et al., 2010). However, these functional studies can be performed in future through site directed mutagenesis or CRISPR-Cas9 technology to gain more knowledge on the effect of these mutations pertaining to the mitochondrial functions. Besides that, mitochondria proteomics would be another field of interest that might provide more understanding on the true protein outcome of mtDNA defects detected or predicted from non-synonymous mutations (Ordys et al., 2010). Finally, the potential mutations could be due to cell culture conditions instead of glioma. Hence, it would be ideal to use patient-derived tumor and matched normal tissues, which are available through many tumor tissue banks.

mtDNA mutations have been identified in many types of cancers including colorectal, bladder, breast, lung, thyroid, and prostate as well as glioma (Kuo et al., 2010; Zhang et al., 2010, 2015; Cui et al., 2013). The translational impact to look at diagnostic and prognostic values of mtDNA mutations was also studied. However, the results were considered controversial for glioma where some studied showed positive outcome (Mohamed Yusoff, 2017). For example, Zhang and colleagues showed that glioma patients with high mtDNA content have increased survival time compared to patients with low mtDNA content. On the other hand, other authors suggested that mtDNA mutation is not useful as predictive biomarkers for diagnostics and prognostics of glioma. Studies in a larger sample size are needed to further evaluate the usefulness of mtDNA mutations as molecular biomarkers for glioma.

## Conclusion

In conclusion, our study showed that Grade II 1321N1 and Grade III SW1783 cell lines harbored eloquent coding region mtDNA mutations that were predicted to be harmful to their respiratory complex functions. Functional analysis proved that these cells had poorer mitochondrial function and 1321N1 cells also displayed high oxidative stress. Grade IV cells, surprisingly, did not show any non-synonymous mutation. In fact, they had the highest antioxidant ability and its cause remains to be further elucidated. These findings warrant further confirmation using variable grades of glioma tissue samples. However, mtDNA mutations are clearly involved in tumorigenesis. Nonetheless, their roles in cancer development, whether merely being passenger mutations, arising by chance, or whether they could significantly contribute to the initiation and promotion of tumor, is still a debatable and challenging question.

## Author contributions

BS, Experiment design, laboratory work, data analysis, manuscript writing. NA, Laboratory work supervision, data analysis, and manuscript correction. ST, Experiment design, laboratory work supervision and data analysis. AA, FF, JT, MM, TC, Sample collection and critical comments on manuscript. AM, Statistical analysis supervision. RH, WW, Data analysis supervision and manuscript correction. RJ, Project leader, principal investigator, experiment design, data analysis supervision, and manuscript correction.

## Funding

This study was supported by Higher Institution Centre of Excellence grant from the Ministry of Higher Education Malaysia (Grant number: AKU49).

### Conflict of interest statement

The authors declare that the research was conducted in the absence of any commercial or financial relationships that could be construed as a potential conflict of interest. The reviewers IH and FC and handling Editor declared their shared affiliation, and the handling Editor states that the process nevertheless met the standards of a fair and objective review.
